# Human Placenta Hydrolysate Protects Against Acetaminophen-Induced Liver Injury in Mice

**DOI:** 10.3390/biomedicines13051219

**Published:** 2025-05-18

**Authors:** Inyoung Hwang, Chi-Gu Kang, So-Jung Lim, Hyun-Jin Kim, Ryun Kang, So-Hyun Jeon, Sang-Hoon Lee, Jae-Won Kim, Ju-Seop Kang

**Affiliations:** 1 Department of Clinical Pharmacology and Therapeutics, Hanyang University Seoul Hospital, Seoul 04736, Republic of Korea; isoleucine@hanyang.ac.kr; 2 Department of Pharmacology, College of Medicine, Hanyang University, Seoul 04763, Republic of Korea; 3 Department of Medical and Digital Engineering, College of Engineering, Hanyang University, Seoul 04736, Republic of Korea; z2021113673@hanyang.ac.kr; 4 Research and Development Center, Green Cross Wellbeing Corporation, Yongin 16950, Republic of Korea; sh.jeon@gccorp.com (S.-H.J.); kimphage@gccorp.com (J.-W.K.); 5 Department of Family Medicine and Functional Medicine, Green Cross I-MED Gangnam Center, Seoul 06655, Republic of Korea; shlee0612@gccorp.com

**Keywords:** acetaminophen, drug-induced liver injury, human placenta hydrolysate, antioxidant, anti-inflammatory agent

## Abstract

**Background/Objectives**: Acetaminophen (APAP) is a widely used analgesic and antipyretic, but overdose can lead to APAP-induced liver injury (AILI), a major cause of acute liver failure. While N-acetylcysteine (NAC) is the current standard of care, its efficacy is significantly reduced when administered after the peak time of liver injury, highlighting the need for alternative therapeutic strategies. Human placenta hydrolysate (HPH) has shown potential as a therapeutic agent for various liver diseases due to its rich content of bioactive compounds. This study aimed to investigate the hepatoprotective effects of HPH in a mouse model of AILI. **Methods**: HPH was administered to mice for three days prior to APAP treatment. The effects of HPH on liver morphology, necrosis, liver enzymes, phase I/II detoxification enzymes, oxidative stress markers, and inflammatory cytokines were evaluated. **Results**: HPH pretreatment attenuated APAP-induced liver necrosis and congestion, reduced serum levels of liver enzymes. In addition, HPH showed a concentration-dependent attenuation of APAP-induced decrease in human hepatocyte viability. HPH modulated phase I/II enzyme expression by downregulating CYP2E1 and upregulating SULT1A1, UGT1A6, GSTP1, and TPMT. HPH also exhibited antioxidant effects by increasing SOD and GPx activities, reducing MDA levels, and restoring the GSH/GSSG ratio. Furthermore, HPH attenuated the APAP-induced increase in the inflammatory cytokines TNF-α and IL-6. These findings suggest that HPH protects against AILI through multiple mechanisms, including the modulation of phase I/II detoxification, activation of antioxidants, and inhibition of inflammation. **Conclusions**: HPH could be a potential therapeutic option for APAP overdose and related liver injuries.

## 1. Introduction

Acetaminophen (APAP) is one of the most widely used over-the-counter analgesics and antipyretics. Although APAP is regarded as safe within the recommended doses, overdose can lead to APAP-induced liver injury (AILI), which is considered the leading cause of acute liver failure (ALF) in many countries [1]. At therapeutic doses, most APAP is converted to non-toxic adducts by phase II conjugating enzymes, mainly UDP-glucuronosyltransferase (UGT) and sulfotransferase (SULT), and excreted in the urine. A minor portion is metabolized by cytochrome P450 enzymes (CYPs), particularly CYP2E1, to form the highly reactive metabolite N-acetyl-p-benzoquinone imine (NAPQI) [2]. Under normal physiological conditions, NAPQI is rapidly detoxified by conjugation with glutathione (GSH). However, at toxic doses, phase II enzymes become saturated and the overproduction of NAPQI depletes GSH, causing oxidative stress, mitochondrial dysfunction, and hepatocyte necrosis [3].

Because a significant number of patients with ALF secondary to AILI undergo liver transplantation and have a high mortality rate, additional therapeutic options are necessary [4,5]. Currently, N-acetylcysteine (NAC) is the only therapeutic option for patients with APAP overdose [6]. Administration of NAC, an antioxidant, at the early stage of AILI can replenish the depleted GSH pool and prevent the accumulation of the noxious metabolite NAPQI [7,8]. However, NAC is most effective if administered within 8 h of APAP overdose [9,10]. Unfortunately, most APAP overdose patients receive treatment after the peak time of liver injury, when the effectiveness of NAC has significantly declined [9,10]. Therefore, there is a need for alternative hepatoprotective therapeutic options that are effective during the metabolism of APAP and liver injury.

Human placenta hydrolysate (HPH) is a rich source of bioactive compounds, including amino acids, peptides, cytokines, and growth factors [11,12,13]. HPH has been shown to promote liver regeneration by activating cytokines and growth factors and reducing oxidative stress [14]. Moreover, HPH has shown anti-apoptotic effects against hepatocyte toxicity both in vivo and in vitro [15]. Because these effects are mediated by supplementation with GSH and its components, along with the activation of antioxidative enzymes such as superoxide dismutase (SOD) and glutathione peroxidase (GPx), HPH could be considered a candidate therapeutic option for AILI. 

This study aimed to evaluate the hepatoprotective effects of HPH in a mouse model of AILI. By analyzing the influence of HPH on phase I and II detoxification enzymes, oxidative stress markers, and hepatocellular damage, this study sought to determine whether HPH could serve as a novel therapeutic approach for AILI. 

## 2. Materials and Methods

### 2.1. Preparation of Human Placenta Hydrolysate

HPH (Laennec^®^, Green Cross WellBeing Corp., Seoul, Republic of Korea) was prepared by hydrolysis of the human placenta using HCl and pepsin. The final product was in the liquid form. 

### 2.2. In Vitro Cell Viability Assay

Cryopreserved primary human hepatocytes (LIFEHEPA^®^ plateable hepatocytes) supplied by HLB Cell Co. Ltd. (Dongtan, Republic of Korea) were thawed and seeded onto collagen type I-coated 12-well plate (SPLCoat^TM^, SPL Life Sciences Co. Ltd., Pocheon, Republic of Korea) at an appropriate density. The hepatocytes were maintained in Williams’ Medium E, Modified (LM017-02, Welgene Inc., Gyeongsan, Republic of Korea) with 10% FBS and 1% penicillin/streptomycin. After 24 h, the wells were divided into six groups to receive respective treatment: (a) Normal (no treatment), (b) Control (APAP 20 mM), (c) NAC (N-acetylcysteine 10 mM for one hour followed by APAP 20 mM), (d) HPH 5% (Laennec^®^ 5% for two hours followed by APAP 20 mM), (e) HPH 10% (Laennec^®^ 10% for two hours followed by APAP 20 mM), and (f) HPH 15% (Laennec^®^ 15% for two hours followed by APAP 20 mM). MTT (Thiazolyl Blue Tetrazolium Bromide, Sigma-Aldrich (Saint Louis, MO, USA), CAS-No: 293-93-1) assay was performed for each well to assess cell viability.

### 2.3. Animals and Experimental Design

Six-week-old male C57BL mice were supplied by Raonbio, Inc. (Yongin, Republic of Korea). During the experimental period, mice were provided with adequate food and water *ad libitum*. The animal room was maintained at a temperature of 22 ± 2 °C and humidity of 50 ± 20%, with a 12-h light/dark cycle. After acclimation and weight measurement, the mice divided into five groups and were administered the respective test substance once a day for three days: (a) normal (PBS 200 μL), (b) control (PBS 200 μL), (c) HPH (Laennec^®^ 8.2 mL/kg, 200 μL), (d) UDCA (ursodeoxycholic acid 250 mg/kg), and (e) NAC (NAC 100 mg/kg). PBS and HPH were administered via intraperitoneal injection, whereas UDCA and NAC were administered orally. Following an overnight fast, all groups, except the normal group, received APAP (500 mg/kg, 200 μL) dissolved in PBS warmed to approximately 55 °C to ensure complete dissolution and subsequently cooled to room temperature prior to injection, intraperitoneally one hour after the last administration of the test substance. Necropsy was performed 24 h after APAP administration to collect blood and liver tissue. Blood samples were collected from the inferior vena cava during the necropsy. After incubation at room temperature for 1.5 h to ensure complete clot formation, the serum samples were separated from the blood samples by centrifugation (1000× *g*, 15 min, 4 °C) and stored in a deep freezer. Liver tissues were harvested after PBS perfusion during necropsy and were stored in a deep freezer.

### 2.4. Enzyme-Linked Immunosorbent Assay (ELISA)

ELISA kits (MyBioSource, San Diego, CA, USA) were used to measure the activities or levels of the following enzymes and biomarkers: phase I enzyme (CYP2E1), antioxidants (SOD, Malondialdehyde (MDA), GSH), phase II enzymes (SULT1A1, UGT1A6, glutathione S-transferase P1 (GSTP1), thiopurine methyltransferase (TPMT)), and liver enzymes (aspartate aminotransferase (AST) and alanine aminotransferase (ALT)). The assay was conducted using mouse serum or liver tissue following the protocol provided in the ELISA kit. The optical density (OD) of each well was measured using a spectrometer (SpectraMax i3x, Molecular Devices, San Jose, CA, USA).

### 2.5. Quantitative Real-Time Polymerase Chain Reaction (RT-PCR)

The liver tissue harvested during necropsy was cut into approximately 30 mg pieces. After transfer to a mortar, the tissue was ground into powder with the addition of liquid nitrogen. Total RNA was extracted using the RNeasy Mini Kit (Qiagen, Hilden, Germany), according to the manufacturer’s protocol. A reverse transcription kit (Applied Biosystems, Foster City, CA, USA) was used to synthesize cDNA from 1 μg of the extracted RNA. The PCR reaction mixture contained 2 μL cDNA (10 ng), 10 μL SYBR Green, 0.5 μL 10 μM forward/reverse primer, and 7 μL distilled water. RT-PCR was performed using the QuantStudio 3 (Applied Biosystems, Foster City, CA, USA). Expression data were calculated using the quantification method based on ΔΔCt values.

### 2.6. Statistical Analysis

Data are presented as mean ± standard deviation. Statistical comparisons between groups were performed using a one-way analysis of variance (ANOVA) followed by Fisher’s Least Significant Difference (LSD) post hoc test when the assumption of normality was met, as assessed by Shapiro-Wilk test. For data that did not meet the normality assumption, the Kruskal-Wallis test, followed by Dwass-Steel-Critchlow-Fligner (DSCF) multiple comparison test, was utilized as a non-parametric alternative. All statistical analyses were conducted using SAS version 9.4 (SAS Institute, Cary, NC, USA). Statistical significance was set at *p* < 0.05.

## 3. Results

### 3.1. Effect of HPH on In Vitro Hepatocyte Viability

**Figure 1:**
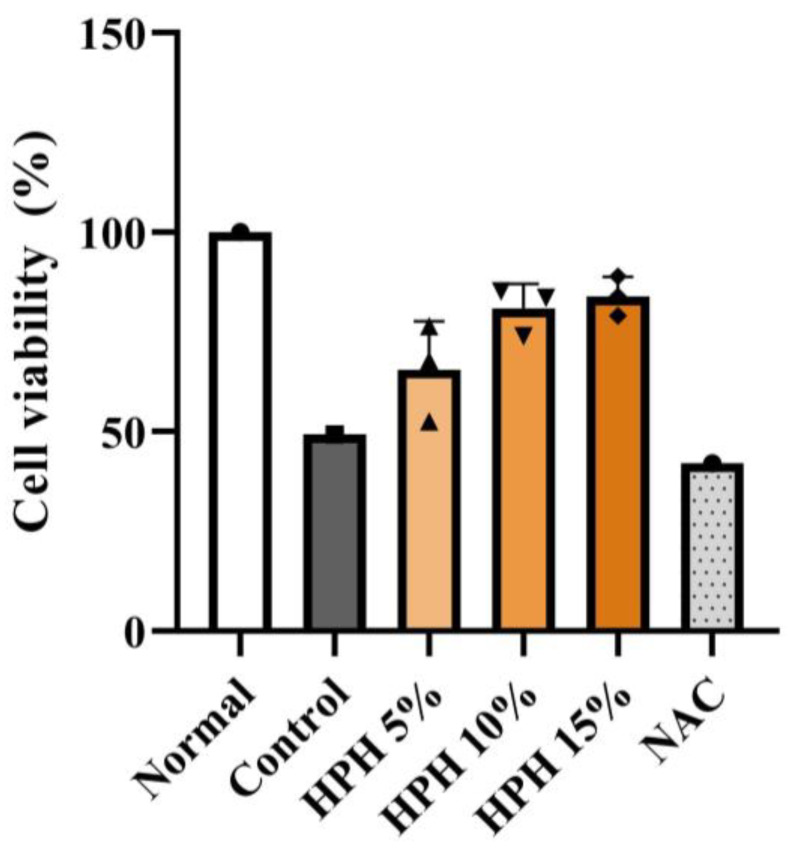
Effect of HPH on in vitro viability of human hepatocytes. N = 1 for the normal, control, and NAC groups. N = 3 for HPH 5%, 10%, and 15% groups.

Although formal statistical comparisons between groups were not conducted owing to the small sample size, the HPH groups showed a tendency to attenuate the decrease in cell viability by APAP. This attenuation was more profound at higher concentrations of HPH (Figure 1).

### 3.2. Effect of HPH on APAP-Induced Acute Liver Injury

**Figure 2:**
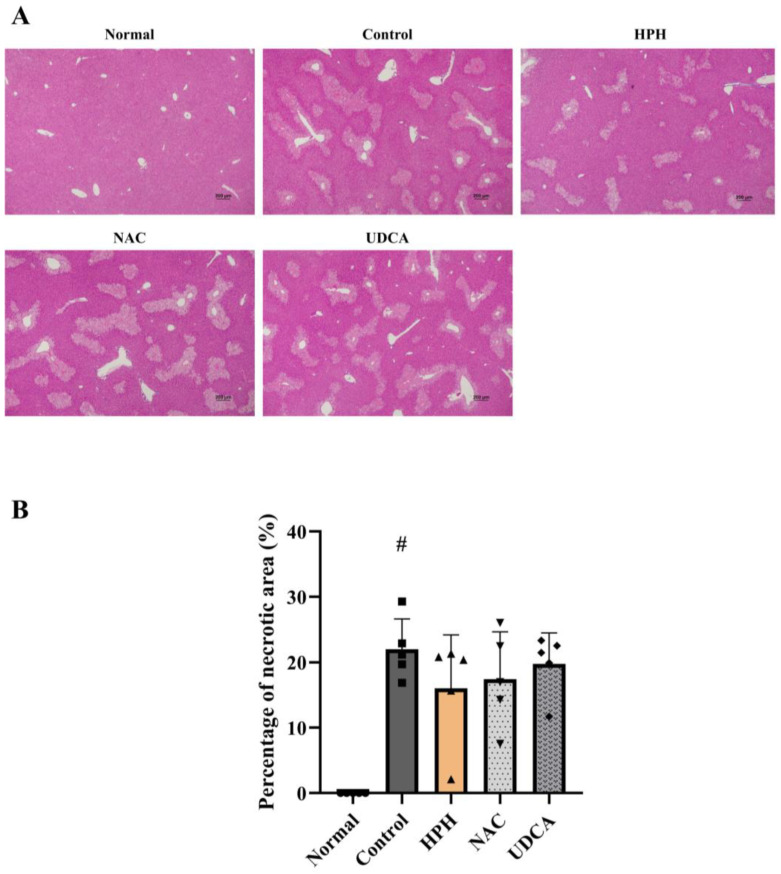
Effects of HPH on the necrotic area of liver tissue in AILI mice: (**A**) H&E staining of liver tissue (×50); (**B**) percentage of necrotic area of liver tissue. N = 5 per group. # *p* < 0.05 versus normal.

APAP-induced hepatotoxicity leads to congestion, hemorrhage, and necrosis of the liver parenchyma [16,17]. In contrast to the normal group, the liver tissues of the control group treated with APAP exhibited widespread necrotic spots, which appeared as white necrotic patches, along with severe congestion, characterized by dark discoloration. The HPH group showed mild necrosis and congestion compared with the control group. Similarly, the NAC and UDCA groups showed mild necrosis and congestion, comparable to those observed in the HPH group (Figure S1). Supporting these macroscopic observations, Hematoxylin and Eosin (H&E) staining of liver tissue showed 22.0% necrotic area in the APAP-treated control group, whereas no necrotic area was found in the normal group. Although not statistically significant, HPH reduced the necrotic area to 16.1%, while NAC and UDCA decreased the necrotic area to 17.5% and 19.8%, respectively (Figure 2A,B). High-power magnification (×400) of H&E-stained sections confirmed these findings, predominantly revealing centrilobular necrosis, a characteristic feature of drug-induced liver injury (Figure S2). 

### 3.3. Effect of HPH on Liver Enzymes

**Figure 3:**
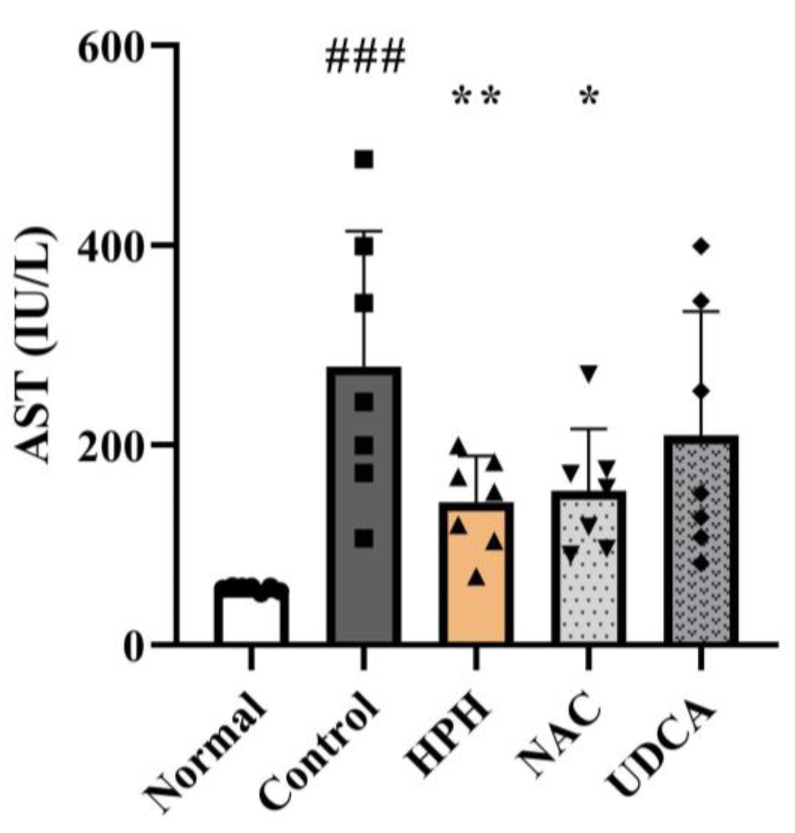
Effects of HPH on AST in AILI mice. N = 7 per group. ### *p* < 0.001 versus normal. * *p* < 0.05, ** *p* < 0.01 versus control.

AST and ALT are liver enzymes released from damaged hepatocytes into the bloodstream when the liver is injured, making them sensitive indicators of hepatocellular damage [18]. The serum levels of AST and ALT in the APAP-treated control group increased significantly by 4.9-fold and 35.5-fold, respectively, compared to those in the normal group. In contrast, the AST and ALT levels decreased by 49.4% (statistically significant) and 37.5%, respectively, in the HPH group. The NAC group showed a comparable reduction in the liver enzyme levels, whereas the UDCA group showed a smaller reduction (Figure 3 and Figure S3).

### 3.4. Effect of HPH on Modulation of Phase Ⅰ/Ⅱ Enzyme Expression

**Figure 4:**
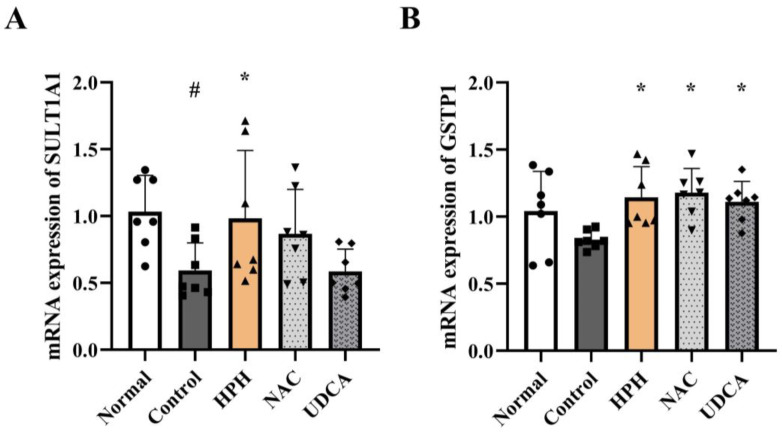
Effects of HPH on hepatic phase II enzyme expression level in AILI mice: (**A**) SULT1A1; (**B**) GSTP1. N = 7 per group. # *p* < 0.05 versus normal. * *p* < 0.05 versus control.

Approximately 90% of APAP is metabolized in the liver via phase II enzymes into sulfate and glucuronide conjugates, which are excreted in the urine, whereas the remainder is converted into the potentially toxic NAPQI via phase I reactions, mainly by CYP2E1 [4]. APAP treatment in the control group resulted in increased expression of CYP2E1. Administration of HPH reduced the expression of CYP2E1 to levels similar to those in the normal group, although the difference was not statistically significant. UDCA and NAC showed comparable reduction (Figure S4A). In the case of phase II reactions, the expression of the APAP-metabolizing enzymes SULT1A1 and UGT1A6, and TPMT was significantly decreased in the APAP-treated control group. In contrast, in the HPH group, the expression of SULT1A1 and GSTP1 increased significantly by 65.6% and 38.1%, respectively, compared to the control group (Figure 4A,B). The expression of UGT1A6 and TPMT in the HPH group also increased by 61.5% and 21.2%, respectively, although this increase was not statistically significant (Figure S4B,C). 

### 3.5. Effect of HPH on Antioxidant Activity

**Figure 5:**
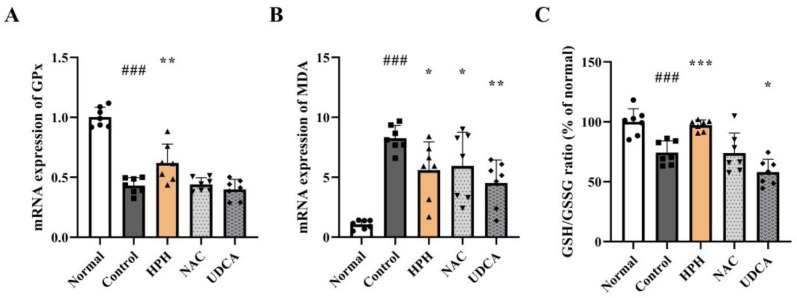
Effects of HPH on antioxidant activity in AILI mice: (**A**) GPx; (**B**) MDA; (**C**) GSH/GSSG ratio. N = 7 per group. ### *p* < 0.001 versus normal. * *p* < 0.05, ** *p* < 0.01, *** *p* < 0.001 versus control.

Excessive NAPQI formation following APAP overdose leads to GSH depletion and adduct formation of mitochondrial proteins, resulting in mitochondrial dysfunction and generation of reactive oxygens species (ROS) [19]. The activity of the antioxidant enzyme GPx decreased in the APAP-treated control group compared to the normal group. In contrast, the HPH group exhibited a 43.7% increase in GPx expression relative to the control group (Figure 5A). SOD, another antioxidant enzyme, showed a similar tendency; however, the differences between the groups were not statistically significant (Figure S5). In addition, the expression of MDA, a lipid peroxidation metabolite used as a marker for intracellular oxidative stress [20], showed an eight-fold increase in the APAP-treated control group compared to the normal group. Conversely, the HPH group showed a significant reduction (32.3%) in MDA expression compared to the control group (Figure 5B). 

Since APAP overdose results in excessive production of NAPQI that overwhelms the hepatic GSH capacity, and the residual NAPQI subsequently causes liver damage, restoring and maintaining hepatic GSH stores is essential for treating AILI [21]. Glutathione exists in reduced (GSH) and oxidized (GSSG) forms, and the intracellular GSH/GSSG ratio serves as an indicator of oxidative stress. In healthy cells, over 90% of the total glutathione is in the reduced form (GSH), with the remainder in the oxidized form (GSSG) [22]. The GSH/GSSG ratio in the APAP-treated control group decreased significantly by 25.7% compared to the normal group. In contrast, the HPH group showed a significant increase (30.8%) in the GSH/GSSG ratio compared to the control group, restoring the levels to those similar to the normal group. Notably, only the HPH group exhibited a statistically significant increase in the GSH/GSSG ratio, whereas the positive control groups, NAC and UDCA, did not (Figure 5C).

### 3.6. Effect of HPH on Inflammatory Cytokines

**Figure 6:**
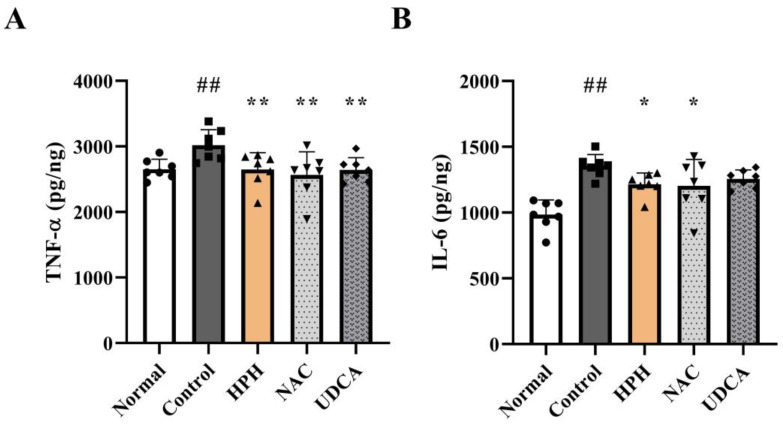
Effects of HPH on inflammatory cytokines in AILI mice: (**A**) TNF-α; (**B**) IL-6. N = 7 per group. ## *p* < 0.01 versus normal. * *p* < 0.05, ** *p* < 0.01 versus control.

In AILI, necrotic hepatocytes release various damage-associated molecular patterns (DAMPs) to activate the innate immune response and promote leukocyte infiltration [23,24]. Upon activation, immune cells release inflammatory cytokines, including TNF-α, IL-1β, and IL-6, which are involved in liver damage [23]. Hepatic concentrations of TNF-α and IL-6 in the APAP-treated control group were significantly higher than those in the normal group. In contrast, the concentrations of TNF-α, IL-1β, and IL-6 in the HPH group decreased significantly by 12.3% and 10.3%, respectively. A comparable decrease was observed in the NAC and UDCA groups (Figure 6A,B). IL-1β also showed a similar pattern, although the differences between the groups were not statistically significant (Figure S6).

## 4. Discussion

Although APAP overdose is a major cause of ALF in many countries, therapeutic options for this condition have limitations [4]. The current study showed that HPH protected the liver of an AILI mouse model via multiple mechanisms, including phase I/II detoxification, antioxidants, and inflammation.

In this study, the reduction of liver injury was assessed by gross morphological changes in the liver, measurement of necrotic area in the liver tissue, alteration of liver enzymes, and in vitro cell viability assay. The livers of the APAP-treated mice exhibited widespread necrotic patches and congestion. The observed morphological changes were attenuated in groups treated with HPH, NAC, and UDCA. HE staining of the liver tissues indicated that APAP-induced necrosis of hepatocytes was reduced in the HPH-treated group, while the NAC and UCDA-treated groups showed lesser extent of decrease, suggesting that the reduction of liver injury was most profound in the HPH group. In line with these findings, an in vitro cell viability assay demonstrated a tendency of HPH to attenuate the decrease in human hepatocyte viability induced by APAP. Although formal statistical comparisons were not conducted owing to the small sample size, the protective effect was more evident at higher concentrations of HPH. The decrease in AST and ALT, well-known markers of hepatocellular injury, was also most profound in the HPH-treated groups compared to the other groups. Similar to our findings, a previous study showed that HPH alleviates hepatotoxin-induced liver injury in vivo and in vitro [25]. Therefore, the hepatoprotective effect of HPH may be applicable to liver injury induced by various hepatotoxins.

HPH is a complex mixture of various bioactive substances, including polydeoxyribonucleotides, RNA, DNA, amino acids and peptides, enzymes, and trace elements [26]. Among these components, certain amino acids have well-documented roles in liver protection. For instance, alanine administration has been reported to decrease ALT and total bilirubin levels in D-galactosamine-treated Sprague-Dawley rat and to prevent both ALT elevation and liver damage in CCL_4_-induced liver injury in rat [27,28]. Glutamate functions as an intracellular antioxidant by contributing to glutathione synthesis [29]. Other amino acids, such as aspartate, glycine, histidine, and serine, have also demonstrated therapeutic effects in in vivo liver disease models [30]. Regarding peptides and growth factors, hepatocyte growth factor (HGF), epidermal growth factor (EGF), and fibroblast growth factor (FGF) have been reported in placental extracts and are recognized for their capacity to induce liver regeneration [31]. Furthermore, HPH is itself a source of various cytokines and has been shown to reduce levels of pro-inflammatory cytokines such as IL-6 and TNF-α [32]. Other important constituents of HPH are nucleic acid derivatives, including polydeoxyribonucleotides (PDRNs), which are known for their tissue repair-promoting and anti-inflammatory properties [26].

Among the targets for the attenuation of AILI, modulation of the phase I/II detoxification process may have beneficial effects. The expression of CYP2E1, a phase I enzyme responsible for the generation of NAPQI, decreased while the expression of SULT1A1, UGT1A6, GSTP1, and TPMT, a phase II metabolizing enzyme, increased in the HPH group compared to the APAP-treated control group. Similarly, a previous study showed that the activation of liver X receptors (LXR) prevents AILI by inducing phase II enzymes and suppressing phase I enzymes [33]. Therefore, alteration of nuclear receptors and transcription factors that regulate the expression and activities of phase I/II enzymes could potentially alter AILI by modulating the generation of NAPQI [2].

Oxidative stress is another key step in APAP-induced hepatotoxicity. Moreover, the recovery of the GSH/GSSG ratio comparable to that of the normal group in the HPH group indicated that the GSH pool was restored to prevent further damage caused by oxidative stress. Such antioxidative properties have been shown by various components of placental extract, such as uracil, L-tyrosine, L-phenylalanine, L-tryptophan, and collagen peptides [34]. The mechanism responsible for the antioxidant effect of HPH involves the Nrf2 pathway, which increases antioxidant enzyme and hepatic GSH levels [15,34], as shown in this study.

Necrosis of hepatocytes releases various DAMPs into the extracellular space, stimulating the production of inflammatory mediators by immune cells [2]. Kupffer cells (KCs), the resident hepatic macrophages, are among the first responders and recognize DAMPs via pattern recognition receptors such as Toll-like receptors (TLRs), leading to their activation [35]. Activated KCs are a primary source of TNF-α in the early phase of AILI [35]. TNF-α plays a critical role by sensitizing hepatocytes to apoptosis, promoting further inflammation, and contributing to liver damage [35]. IL-6, secreted by KCs and infiltrating monocytes and macrophages, contributes to the acute phase inflammatory response, and in later stages of injury resolution, to regeneration [36]. In the current study, the increased levels of TNF-α and IL-6 were attenuated in the HPH-treated group. Reduction of macrophage accumulation and inflammatory cytokines by placental extract has been reported in previous studies [15,25,37]. Therefore, the anti-inflammatory effect of HPH may have contributed to the reduction of APAP-induced liver injury.

The current study has several limitations that should be acknowledged. Firstly, primary pathologic endpoint was the necrotic area quantified in H&E-stained liver sections. Future research could employ additional methods, such as the terminal deoxynucleotidyl transferase (dUTP) nick end labeling (TUNEL) assay and immunohistochemistry, to further elucidate the detailed mechanism of cell death and enable more precise quantification of the necrotic area. Secondly, this study evaluated the hepatoprotective effect of HPH and other comparators by administering them before APAP exposure. To assess the therapeutic potential of HPH and the comparators on established AILI, future studies should involve their administration after APAP intoxication.

## 5. Conclusions

In conclusion, this study demonstrated that HPH protected the liver of an APAP-induced hepatitis mouse model via modulation of phase I/II detoxification, activation of antioxidants, and inhibition of inflammation. These results suggest that HPH could be a potential therapeutic option for APAP overdoses or various hepatotoxic situations.

## Data Availability

All data supporting the findings of this study are available within the paper.

## Supplementary Materials

The following supporting information can be downloaded at: https://www.mdpi.com/article/10.3390/biomedicines13051219/s1, Figure S1: Gross morphological changes in the liver. White circles denote representative areas with pathologic features; Figure S2: H&E staining of liver tissue (×400); Figure S3: Effects of HPH on ALT in AILI mice. N = 7 per group. # *p* < 0.05 versus normal; Figure S4: Effects of HPH on hepatic phase II enzyme expression level in AILI mice: (A) CYP2E1; (B) UGT1A6; (C) TPMT. N = 7 per group. # *p* < 0.05, ## *p* < 0.01 versus normal; Figure S5: Effects of HPH on SOD in AILI mice. N = 7 per group; Figure S6: Effects of HPH on IL-1β in AILI mice. N = 7 per group.

## References

[1] Lee W.M. (2013). Drug-induced acute liver failure. Clin. Liver Dis..

[2] Yan M., Huo Y., Yin S., Hu H. (2018). Mechanisms of acetaminophen-induced liver injury and its implications for therapeutic interventions. Redox Biol..

[3] Woolbright B.L., Jaeschke H., Ding W.-X., Yin X.-M. (2017). Mechanisms of Acetaminophen-Induced Liver Injury. Cellular Injury in Liver Diseases.

[4] Yoon E., Babar A., Choudhary M., Kutner M., Pyrsopoulos N. (2016). Acetaminophen-Induced Hepatotoxicity: A Comprehensive Update. J. Clin. Transl. Hepatol..

[5] Bunchorntavakul C., Reddy K.R. (2013). Acetaminophen-related hepatotoxicity. Clin. Liver Dis..

[6] Akakpo J.Y., Ramachandran A., Jaeschke H. (2020). Novel strategies for the treatment of acetaminophen hepatotoxicity. Expert Opin. Drug Metab. Toxicol..

[7] Corcoran G.B., Wong B.K. (1986). Role of glutathione in prevention of acetaminophen-induced hepatotoxicity by N-acetyl-L-cysteine in vivo: Studies with N-acetyl-D-cysteine in mice. J. Pharmacol. Exp. Ther..

[8] Corcoran G.B., Todd E.L., Racz W.J., Hughes H., Smith C.V., Mitchell J.R. (1985). Effects of N-acetylcysteine on the disposition and metabolism of acetaminophen in mice. J. Pharmacol. Exp. Ther..

[9] Smilkstein M.J., Knapp G.L., Kulig K.W., Rumack B.H. (1988). Efficacy of oral N-acetylcysteine in the treatment of acetaminophen overdose. Analysis of the national multicenter study (1976 to 1985). N. Engl. J. Med..

[10] Whyte A.J., Kehrl T., Brooks D.E., Katz K.D., Sokolowski D. (2010). Safety and effectiveness of acetadote for acetaminophen toxicity. J. Emerg. Med..

[11] Jung J., Lee H.J., Lee J.M., Na K.H., Hwang S.G., Kim G.J. (2011). Placenta extract promote liver regeneration in CCl4-injured liver rat model. Int. Immunopharmacol..

[12] Jung J., Moon J.W., Choi J.H., Lee Y.W., Park S.H., Kim G.J. (2015). Epigenetic Alterations of IL-6/STAT3 Signaling by Placental Stem Cells Promote Hepatic Regeneration in a Rat Model with CCl4-induced Liver Injury. Int. J. Stem Cells.

[13] Lee J.O., Jang Y., Park A.Y., Lee J.M., Jeong K., Jeon S.H., Jin H., Im M., Kim J.W., Kim B.J. (2024). Human Placenta Extract (HPH) Suppresses Inflammatory Responses in TNF-α/IFN-γ-Stimulated HaCaT Cells and a DNCB Atopic Dermatitis (AD)-Like Mouse Model. J. Microbiol. Biotechnol..

[14] Lee T.H., Park D.S., Jang J.Y., Lee I., Kim J.M., Choi G.S., Oh C.T., Kim J.Y., Han H.J., Han B.S. (2019). Human Placenta Hydrolysate Promotes Liver Regeneration Activation of the Cytokine/Growth Factor-Mediated Pathway and Anti-oxidative Effect. Biol. Pharm. Bull..

[15] Bak D.H., Na J., Choi M.J., Lee B.C., Oh C.T., Kim J.Y., Han H.J., Kim M.J., Kim T.H., Kim B.J. (2018). Anti-apoptotic effects of human placental hydrolysate against hepatocyte toxicity and. Int. J. Mol. Med..

[16] Hinson J.A., Roberts D.W., James L.P. (2010). Mechanisms of acetaminophen-induced liver necrosis. Handb. Exp. Pharmacol..

[17] Muhammad-Azam F., Nur-Fazila S.H., Ain-Fatin R., Mustapha Noordin M., Yimer N. (2019). Histopathological changes of acetaminophen-induced liver injury and subsequent liver regeneration in BALB/C and ICR mice. Vet. World.

[18] Kwo P.Y., Cohen S.M., Lim J.K. (2017). ACG Clinical Guideline: Evaluation of Abnormal Liver Chemistries. Am. J. Gastroenterol..

[19] Du K., Ramachandran A., Jaeschke H. (2016). Oxidative stress during acetaminophen hepatotoxicity: Sources, pathophysiological role and therapeutic potential. Redox Biol..

[20] Del Rio D., Stewart A.J., Pellegrini N. (2005). A review of recent studies on malondialdehyde as toxic molecule and biological marker of oxidative stress. Nutr. Metab. Cardiovasc. Dis..

[21] Heard K.J. (2008). Acetylcysteine for acetaminophen poisoning. N. Engl. J. Med..

[22] Zitka O., Skalickova S., Gumulec J., Masarik M., Adam V., Hubalek J., Trnkova L., Kruseova J., Eckschlager T., Kizek R. (2012). Redox status expressed as GSH:GSSG ratio as a marker for oxidative stress in paediatric tumour patients. Oncol. Lett..

[23] Yang T., Wang H., Wang X., Li J., Jiang L. (2022). The Dual Role of Innate Immune Response in Acetaminophen-Induced Liver Injury. Biology.

[24] Guo H., Chen S., Xie M., Zhou C., Zheng M. (2021). The complex roles of neutrophils in APAP-induced liver injury. Cell Prolif..

[25] Wu J.J., Yang T., Wang C.Y., Liu Q., Yao J.H., Sun H.J., Kaku T., Liu K.X. (2008). Laennec Protects Murine from Concanavalin A-Induced Liver Injury through Inhibition of Inflammatory Reactions and Hepatocyte Apoptosis. Biol. Pharm. Bull..

[26] Tonello G., Daglio M., Zaccarelli N., Sottofattori E., Mazzei M., Balbi A. (1996). Characterization and quantitation of the active polynucleotide fraction (PDRN) from human placenta, a tissue repair stimulating agent. J. Pharm. Biomed. Anal..

[27] Maezono K., Mawatari K., Kajiwara K., Shinkai A., Maki T. (1996). Effect of alanine on D-galactosamine-induced acute liver failure in rats. Hepatology.

[28] Maezono K., Kajiwara K., Mawatari K., Shinkai A., Torii K., Maki T. (1996). Alanine protects liver from injury caused by F-galactosamine and CCl4. Hepatology.

[29] Wu G., Fang Y.Z., Yang S., Lupton J.R., Turner N.D. (2004). Glutathione metabolism and its implications for health. J. Nutr..

[30] Lee D.Y., Kim E.H. (2019). Therapeutic Effects of Amino Acids in Liver Diseases: Current Studies and Future Perspectives. J. Cancer Prev..

[31] Neo S., Makiishi E., Fujimoto A., Hisasue M. (2021). Human placental hydrolysate promotes the long-term culture of hepatocyte-like cells derived from canine bone marrow. J. Vet. Med. Sci..

[32] Pogozhykh O., Prokopyuk V., Figueiredo C., Pogozhykh D. (2018). Placenta and Placental Derivatives in Regenerative Therapies: Experimental Studies, History, and Prospects. Stem Cells Int..

[33] Saini S.P., Zhang B., Niu Y., Jiang M., Gao J., Zhai Y., Hoon Lee J., Uppal H., Tian H., Tortorici M.A. (2011). Activation of liver X receptor increases acetaminophen clearance and prevents its toxicity in mice. Hepatology.

[34] Shen L.H., Fan L., Zhang Y., Zhu Y.K., Zong X.L., Peng G.N., Cao S.Z. (2022). Protective Effect and Mechanism of Placenta Extract on Liver. Nutrients.

[35] Krenkel O., Mossanen J.C., Tacke F. (2014). Immune mechanisms in acetaminophen-induced acute liver failure. Hepatobiliary Surg. Nutr..

[36] Masubuchi Y., Sugiyama S., Horie T. (2009). Th1/Th2 cytokine balance as a determinant of acetaminophen-induced liver injury. Chem. Biol. Interact..

[37] Yamauchi A., Kamiyoshi A., Koyama T., Iinuma N., Yamaguchi S., Miyazaki H., Hirano E., Kaku T., Shindo T. (2017). Placental extract ameliorates non-alcoholic steatohepatitis (NASH) by exerting protective effects on endothelial cells. Heliyon.

