# Supplementary material for: Human Placenta Hydrolysate Protects Against Acetaminophen-Induced Liver Injury in Mice
Source: Biomedicines. 2025 May 18;13(5):1219. doi: 10.3390/biomedicines13051219 (PMC12109462; doi:10.3390/biomedicines13051219)
Supplement: Supplementary file 1 [file biomedicines-13-01219-s001.zip › biomedicines-3609900-supplementary.pdf]

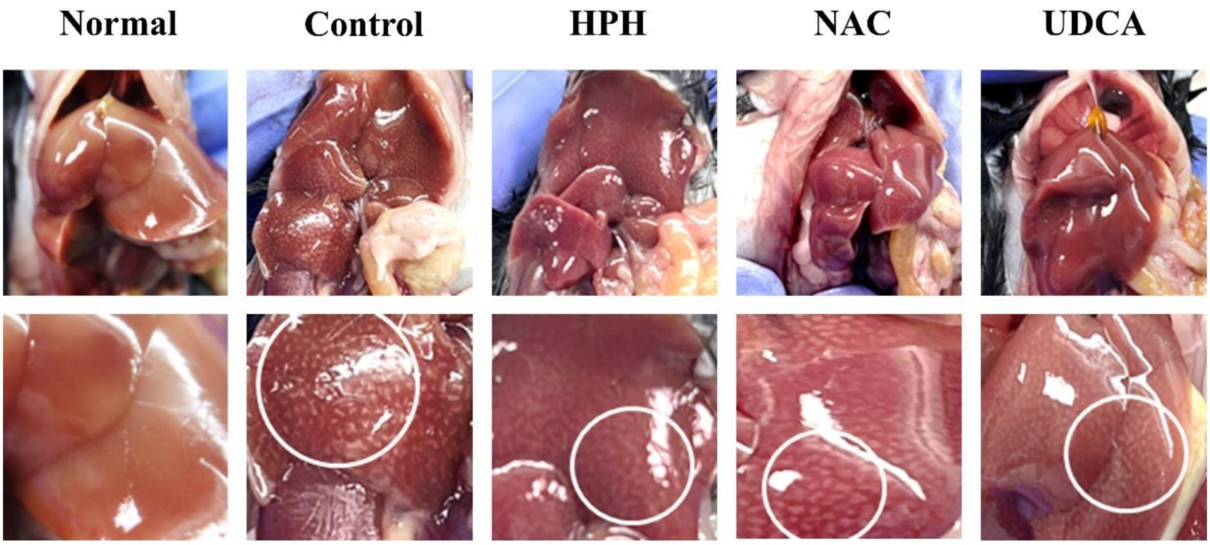

Figure S1. Gross morphological changes in the liver. White circles denote representative areas with pathologic features.

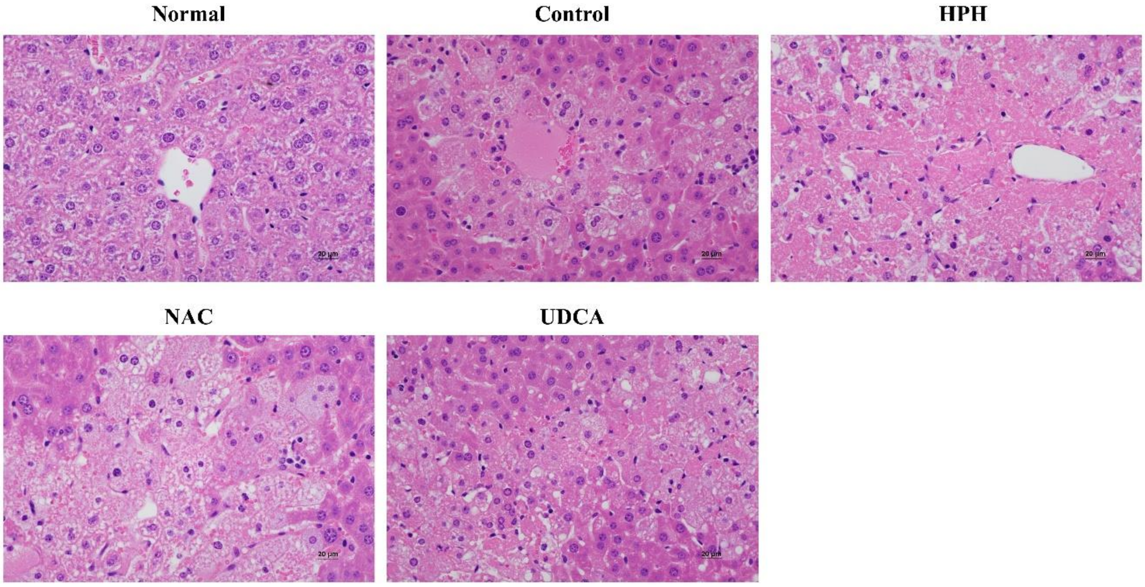

Figure S2. H&E staining of liver tissue (×400)

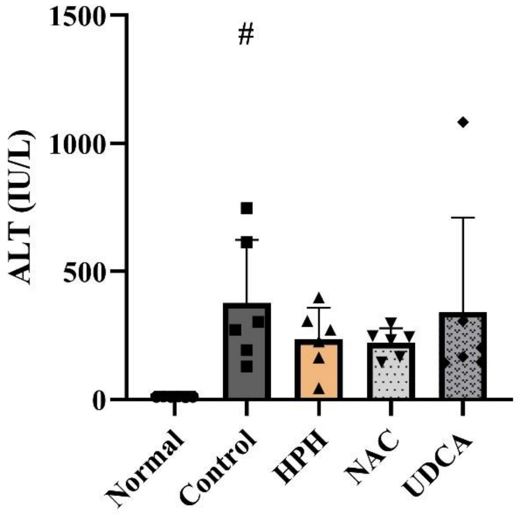

Figure S3. Effects of HPH on ALT in AILI mice. N = 7 per group. #$p < 0.05$ versus normal.

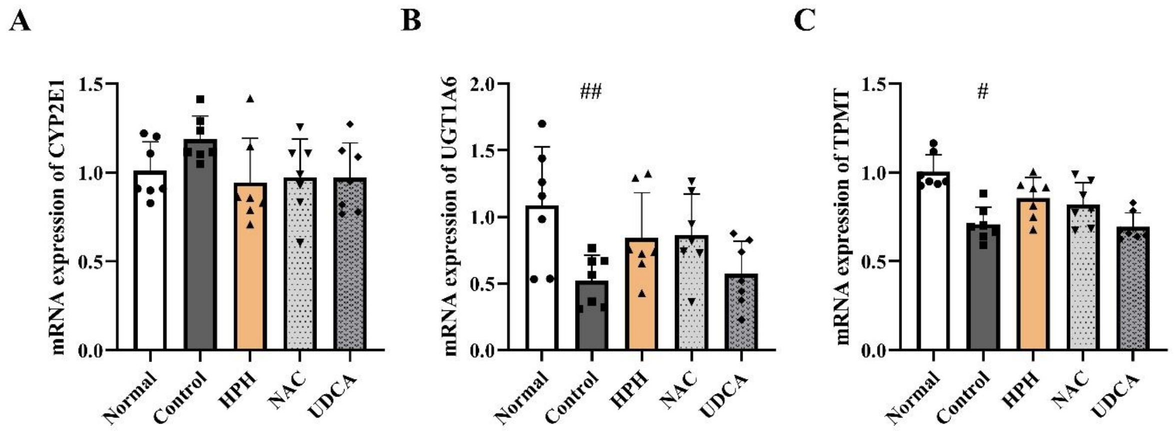

Figure S4. Effects of HPH on hepatic phase II enzyme expression level in AILI mice: (A) CYP2E1; (B) UGT1A6; (C) TPMT. N = 7 per group. #$p < 0.05$, ##$p < 0.01$ versus normal.

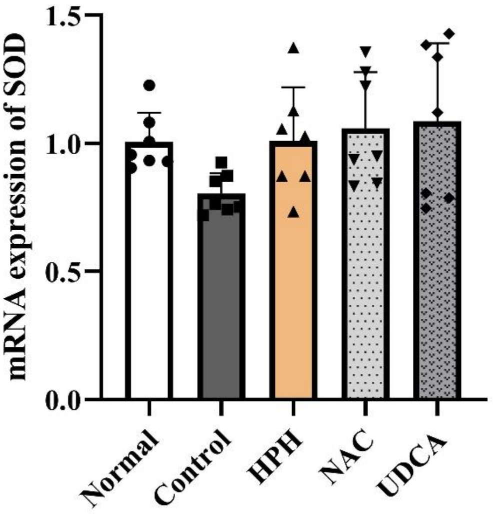

Figure S5. Effects of HPH on SOD in AILI mice. N = 7 per group.

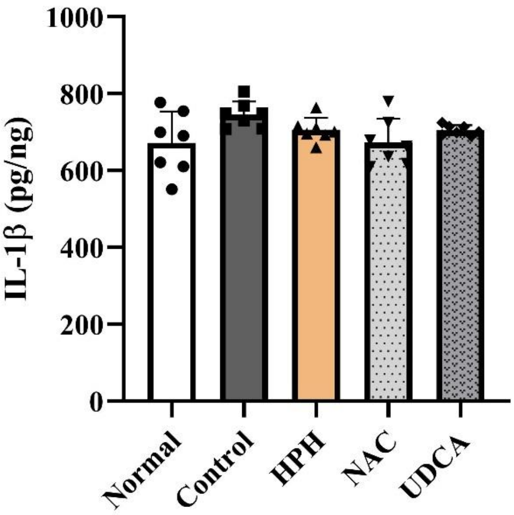

Figure S6. Effects of HPH on IL-1β in AILI mice. N = 7 per group.
